# Integrated analyzes identify CCT3 as a modulator to shape immunosuppressive tumor microenvironment in lung adenocarcinoma

**DOI:** 10.1186/s12885-023-10677-w

**Published:** 2023-03-14

**Authors:** Junfeng Huang, Bingqi Hu, Ying Yang, Huanhuan Liu, Xingyu Fan, Jing Zhou, Liwen Chen

**Affiliations:** 1 Department of Laboratory Medicine, Second Hospital of Anhui Medical University, Hefei, China; 2 Research Center for Translational Medicine, The Second Hospital of Anhui Medical University, Hefei, China

**Keywords:** Lung adenocarcinoma, TCP1, CCT3, Immune cell infiltration, Biomarker

## Abstract

**Background:**

Chaperonin-containing tailless complex polypeptide 1 (TCP1) subunit 3 (CCT3) has tumor-promoting effects in lung adenocarcinoma (LUAD). This study aims to investigate the molecular mechanisms of CCT3 in LUAD oncogenesis.

**Methods:**

The UALCAN databases, Human Protein Atlas (HPA) and The Cancer Genome Atlas (TCGA) data were used to analyze CCT3 expression in LUAD. Both the Wilcoxon rank-sum test and the regression model were used to investigate the connection between clinicopathologic characteristics of LUAD patients and CCT3 expression. The prognostic value of CCT3 was determined by Cox regression models, the Kaplan-Meier method and Nomogram prediction. Next, we identified the most related genes with CCT3 via GeneMANIA and String databases, and the association between CCT3 and infiltrated immune cells using single-sample Gene Set Enrichment Analysis (ssGSEA). CCT3-related pathway enrichment analysis was investigated by GSEA. Finally, CCT3 roles in cell proliferation and apoptosis of LUAD A549 cells was verified by siRNA (small interfering RNA) mediated *CCT3* knockdown.

**Results:**

CCT3 was upregulated in LUAD both in mRNA and protein levels. CCT3 overexpression was associated with clinicopathological characteristics including sex, smoking, T- and N-categories, pathological staging, and a poor prognosis of LUAD patients. GeneMANIA and String databases found a set of CCT3-related genes that are connected to the assembly and stability of proteins involved in proteostasis of cytoskeletal filaments, DNA repair and protein methylation. Furthermore, CCT3 was found to be positively correlated with the infiltrating Th2 cells (r = 0.442, p < 0.01) while negatively correlated with mast cells (r = -0.49, p < 0.01) and immature dendritic cells (iDCs, r = -0.401, p < 0.001) according to ssGSEA analyzes. The pathway analysis based on GSEA method showed that the cell cycle pathway, the protein export pathway, the proteasome pathway and the ribosome pathway are enriched in CCT3 high group, whereas the JAK/STAT pathway, B cell receptor pathway, T cell receptor pathway and toll like receptor pathway were enriched in CCT3 low group. Finally, CCT3 knockdown substantially inhibited proliferation while promoted apoptosis of A549 cells.

**Conclusion:**

Integrated analyzes identify CCT3 as a modulator to shape immunosuppressive tumor microenvironment in LUAD and therefore, a prognostic factor for LUAD.

**Supplementary Information:**

The online version contains supplementary material available at 10.1186/s12885-023-10677-w.

## Introduction

Being the most common type (~ 40%) of lung cancer cases, lung adenocarcinoma (LUAD) is caused by neoplastic distortion of bronchiolar epithelial cells. Multiple molecular-genetic abnormalities have been proposed to be involved in the disease’s pathogenesis and progression, resulting in the diversity and complexity of oncogenic mechanisms that link to poor prognosis of LUAD [1]. Despite significant breakthroughs have been achieved in diagnosis and treatment of LUAD, the disease remains one of the deadliest malignant tumors with an overall survival less than 5 years [2]. Thus, identification of underlying oncogenic mechanisms and therapeutic targets will fuel prospective advances and bring new hope for LUAD patients.

Molecular chaperones act by interacting with, stabilizing and remodeling nonnative polypeptides and directing their substrates into productive folding, transport or degradation pathways. The 60 kDa chaperonin family members consist of molecular chaperones of approximately 60 kDa in size, forming double-ring-shaped protein complexes and exhibiting ATPase activity. Two classes of chaperonins have been defined. Group I comprises bacterial GroEL and its mitochondrial counterpart chaperonin 60 (cpn60) (also known as heat shock protein 60 (Hsp60)) [3]. The chaperonins constituting group II comprise archaebacterial thermosomes and the eukaryotic cytosolic CCT (chaperonin-containing tailless complex polypeptide 1 (TCP1)), which also known as the TCP1 ring complex (TRiC) [4]. CCT/TRiC consists of eight subunits (CCT1-CCT8) and plays a central role in maintaining cellular proteostasis as it mediates the folding of the major cytoskeletal proteins such as tubulins and actins [5].

Of the 8 components consisting of CCT/TRiC complex, CCT3 has been observed to overexpressed in various cancers including LUAD [6–8]. CCT3 suppression led to the inhibition of cell proliferation, cell cycle arrest, cisplatin resistance and ferroptosis/apoptosis of LUAD cells. Signaling molecules including AKT, Yes-associated protein 1 (YAP1), and the Janus kinase 2/signal transducers and activators of transcription 3 (JAK2/STAT3) were reported to be involved in CCT3 effects [7–9]. Furthermore, CCT3 is a critical factor in maintaining intracellular ATP levels and cytoplasmic protein translation in LUAD [10]. Thus, CCT3 is linked to the development and prognosis of LUAD [11–14]. However, there remains a need for more detailed analyses of CCT3-related genetic and protein interaction network and biological pathways in LUAD.

In this study, integrated bioinformatics analyses were performed to investigate CCT3 expression, its predictive value for prognosis, and its related genetic and protein interaction network in LUAD. The association between CCT3 and infiltrating immune cells and biological pathways were also analyzed. Finally, we verified CCT3 effects on cell proliferation and apoptosis of LUAD A549 cells by siRNA mediated *CCT3* knockdown.

## Methods

### Data set acquisition

**Fig. 1:**
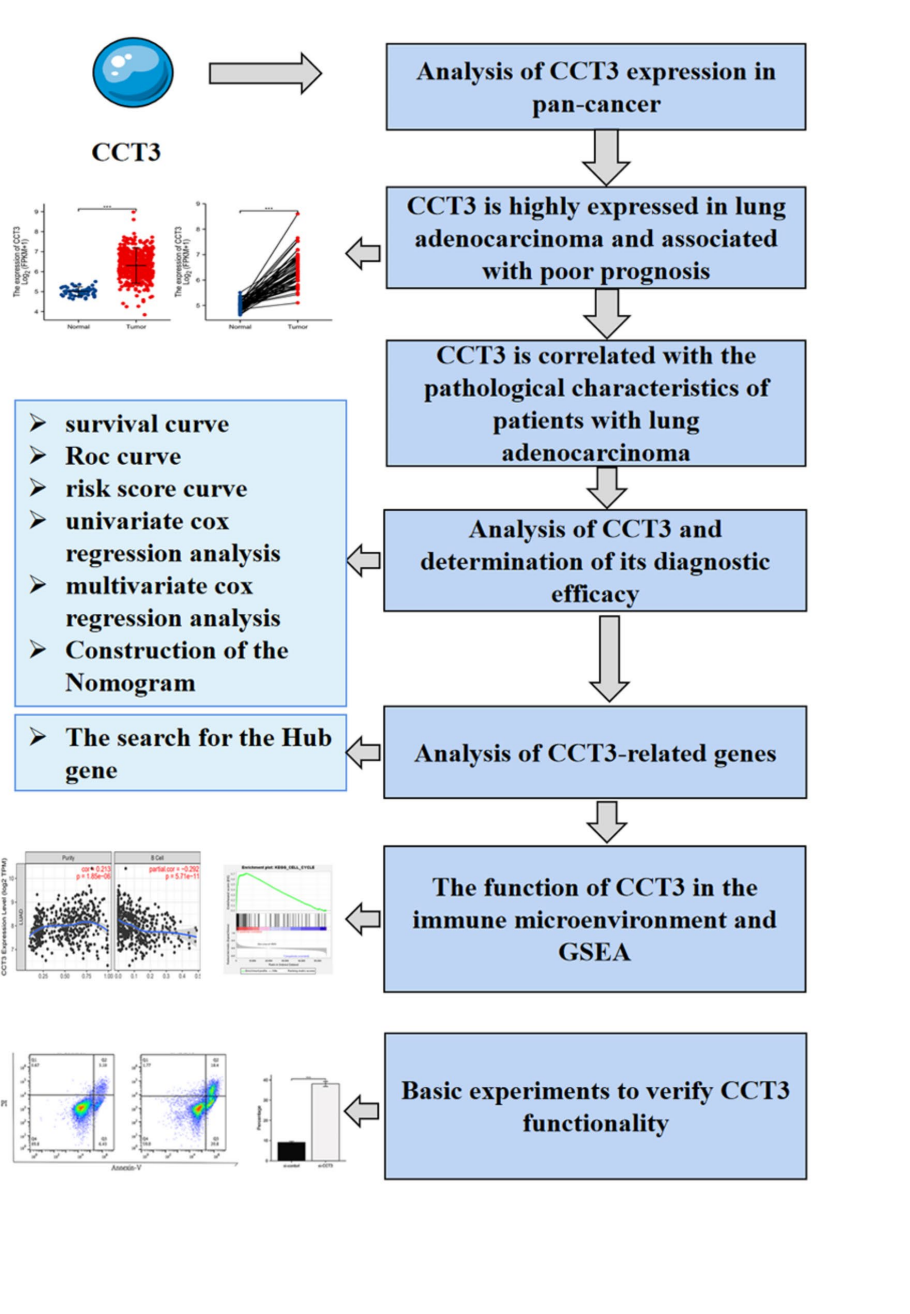
Illustration of the research process

The Cancer Genome Atlas (TCGA) database (The Cancer Genome Atlas Program - National Cancer Institute) was used to gather the high level RNA-Seq data (FPKM, Fragments Per Kilobase of transcript per Million mapped reads) and clinical annotations for LUAD associated genes. Ultimately, 535 LUAD cases and 59 normal cases were included for CCT3 overexpression study. 57 cases with insufficient or missing data were further removed from subsequent survival analysis. The data set used in this study is consistent with the published guidelines of TCGA database, and ethical approval and informed consent are not required. The experimental design of this study is illustrated in Fig. 1.

### The human protein atlas (HPA) database

By using the HPA database (The Human Protein Atlas) [15, 16], we analyzed the expression of CCT3 protein in human normal and tumor tissues in a large series of malignant diseases including LUAD.

### UALCAN [17]

The differentiated expression of CCT3 protein in tumoral and related normal sections of LUAD was verified by the Clinical Proteomic Tumor Analysis Consortium (**CPTAC**) analysis in **UALCAN** (ualcan.path.uab.edu/home) database. Furthermore, the CCT3 mRNA levels between tumor protein (*TP53*) gene mutated and nonmutated LUAD tissues were assessed by the UALCAN database.

### Nomogram generation and univariate/multivariate Cox risk regression analysis

Univariate and multivariate COX regression analysis were performed by using “Survival” package in R (R: The R Project for Statistical Computing (r-project.org)) [18]. Also, “Survival” package was used together with “rms” package to construct the Nomogram.

### The risk score chart construction

The prognostic performance of CCT3 was assessed using a risk score chart plotted by the ggplot2 package.

### The Kaplan-Meier (KM) plotter

KM plotter in GEPIA (Gene Expression Profiling Interactive Analysis) (cancer-pku.cn) Webserver [19]was used to determine the cut-off value of mRNA level in CCT3 survival curve. The logarithmic P-values and 95% confidence interval for the hazard ratio (HR) were calculated and plotted on the graph, and P < 0.05 was considered statistically significant.

### Protein-protein interaction (PPI) network and correlation analysis

GeneMANIA (GeneMANIA) [20]and String (String: functional protein association networks (String-db.org)) [21, 22]were used to construct the PPI network of CCT3 and to seek network-related genes. We further examined the relationship between CCT3 and the hub genes using the heat map (ggplot2 package).

### Systematic analysis of CCT3-related infiltrating immune cells

The composition of CCT3-related infiltrating immune cells was studied by using the single sample Gene Set Enrichment Analysis (ssGSEA).

### GSEA analysis

In this study, GSEA (GSEA (gsea-msigdb.org)) [23]was used to identify classes of genes and proteins associated with high and low expression of CCT3 in LUAD, by using Kyoto Encyclopedia of Genes and Genomes (KEGG) pathway database [24–26]. Samples were dichotomized by the median value of CCT3 mRNA level for GSEA analysis which was carried out a thousand times. P < 0.05 and false discovery rate (FDR) < 0.25 were set as the cutoff criteria.

### Cell culture

The LUAD cell line A549 (cat. no. FH0045) was obtained from the Cell Center of FuHeng Biology (Shanghai, China). Cells were grown in RPMI 1640 (Thermo Fisher Scientific, Inc.) supplemented with 10% FBS (Lonsera Science SRL) and penicillin (100 IU/ml)/streptomycin (100 µg/ml) (MedChemExpress), and in a humidified atmosphere containing 5% CO_2_ at 37˚C. The A549 cell line was authenticated by using short tandem repeat (STR) analysis in combination with sextyping gene amelogenin detection, and was compared with the DSMZ STR cell line profile before use.

**Gene silencing of*CCT3***.

*CCT3* knockdown was performed by transfection of specific small interfering RNAs (siRNAs) using Lipofectamine® 3000 (Thermo Fisher Scientific, Inc.) according to the manufacturer’s protocol. The sequences of the ontarget siRNAs were as follows: siCCT3 #1, 5’GGG ACC ACA UCA GUA AUU ATT-3’ and siCCT3 #2, 5’UAA UUA CUG AUG UGG UCC CTT-3’. Those of the negative control siRNAs oligos were: siControl #1, 5’UUC UCC GAA CGU GUC ACG UTT-3’ and siControl #2, 5’ACG UGA CAC GUU CGG AGA ATT-3’. All the oligos were designed by an online siRNA software, siDirect 2.0 (http://siDirect2.RNAi.jp/), and ordered from Shanghai GenePharma Co., Ltd. The final concentration of Lipofectamine 3000 and siRNAs were 2 µl/ml and 50 nM, respectively. *CCT3* knockdown was verified at 48 h posttransfection via western blotting, as described below.

### Western blotting analysis

In brief, the total cell lysate from siCCT3 and siControl A549 cells were separated via SDS-PAGE, transferred to a polyvinylidene difluoride (PVDF) membrane, and blocked with 5% (w/v) skimmed milk. The primary antibodies against human CCT3 (cat. no. DF12113, 1:1000) and antiGAPDH (cat. no. AF7021, 1:3,000) were from Affinity Biosciences, Ltd. and the secondary antibody HRPconjugated goat antirabbit IgG (H + L) (cat. no. S0001, 1:5,000) was from Affinity Biosciences, Ltd. The expression levels of CCT3 were assessed using ImageJ software (v1.8.0, National Institutes of Health), and were normalized against GAPDH. All experiments were performed in triplicate.

### Colony formation and cell proliferation assay

Cells were seeded in 6-well plates (500 cells per well) and cultured at 37 °C, 5% CO_2_. The medium was changed once 3 days after plating and the colony formation was quantitated 2 weeks later. Briefly, the colonies were fixed with 4% (w/v) paraformaldehyde, stained with 1% crystal violet and counted. Cell proliferation assay was measured in 96-well plate (2000 cells/well with 100 µL) with CCK8 reagent (Dojindo Laboratories, Kumamoto, Japan) as previously described.

### Cell apoptosis analysis

Annexin-V and Propidium Iodide (PI) double staining was conducted via an Annexin-V apoptosis kit (BD, Franklin Lakes, NJ) according to the manufacturer’s instructions for siCCT3 and siControl A549 cells. Cells were analyzed by flow cytometry Epics XL-MCL (Beckman Coulter, CA, USA). Total apoptosis includes Annexin V^+^/PI^−^ (early apoptotic) and Annexin V^+^/PI^+^ (late apoptotic) cells.

### Data statistics and analysis

Data analyzes in this study were done by R4.1.3 and P < 0.05 was considered statistically significant if not otherwise stated. The statistical analysis of the Western blotting and flow cytometry results were performed using t-test and SPSS software version 20.0 (IBM Corp.). Experimental data are displayed as mean with standard deviation (SD) and all the experiments were repeated three times.

## Results

### Pan-cancer and LUAD expression of CCT3 mRNA

**Fig. 2:**
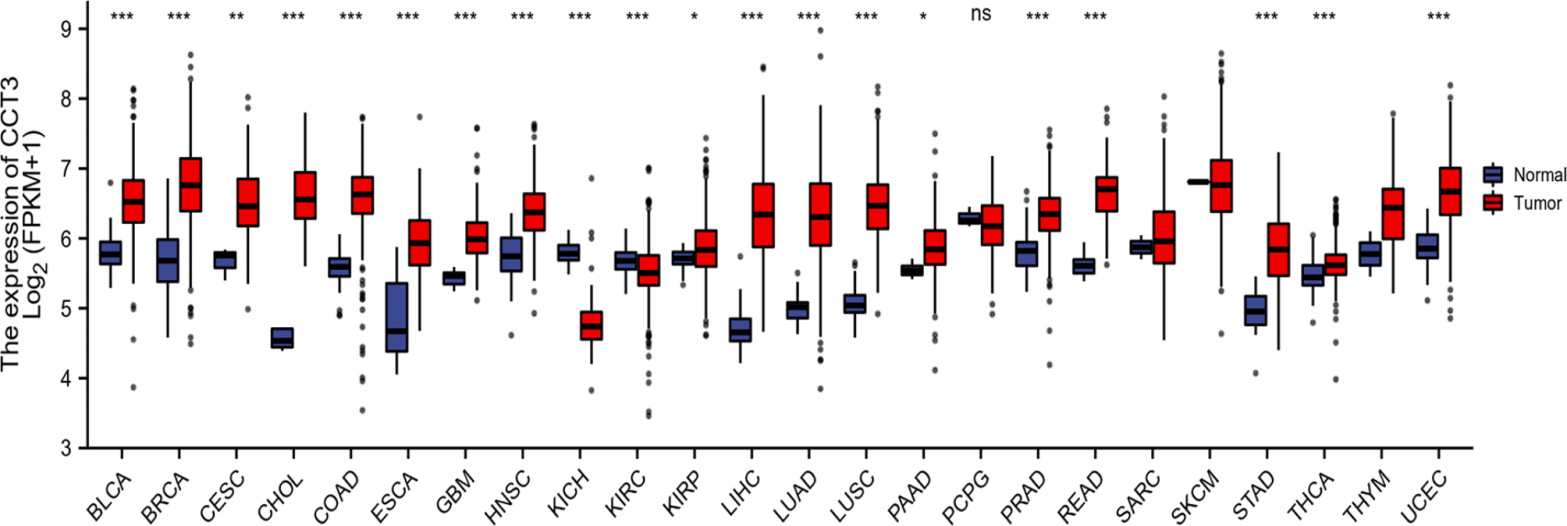
Pan-cancer expression of CCT3 mRNA in TCGA database. *P < 0.05; **P < 0.01; ***P < 0.001; ns, no significance; groups lack statistical analysis means n < 3

**Fig. 3:**
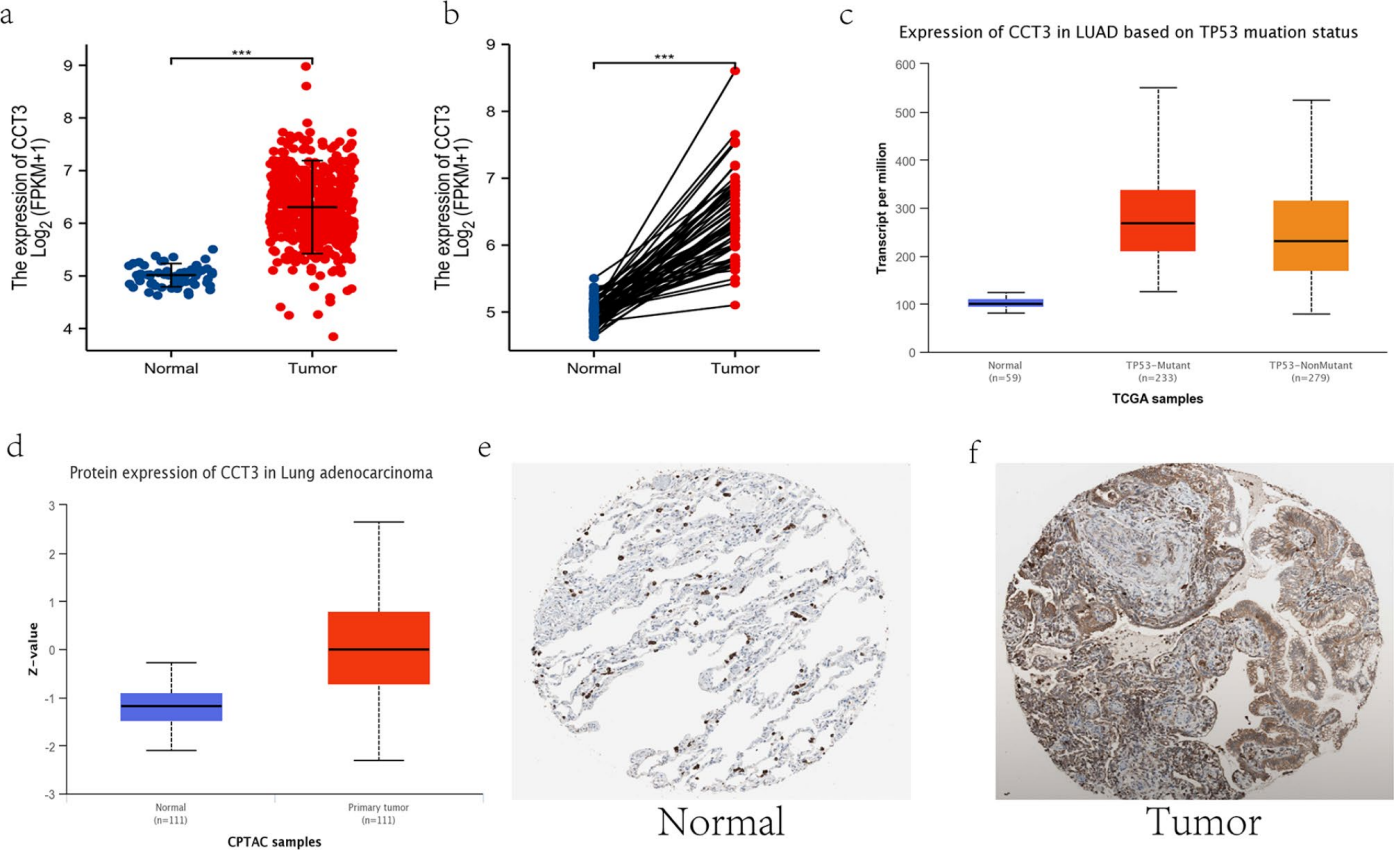
CCT3 expression in LUAD. The expression levels of CCT3 in total **(a)** and paired **(b)** LUAD and normal tissues in TCGA database. Total, Normal = 59 and Tumor = 535; paired, Normal = 57 and Tumor = 57. (**c)** UALCAN database analysis of CCT3 mRNA levels in *TP53*-Mutant, *TP53*-Nonmutant and normal tissues; **(d-f)** Comparison of tumoral and normal CCT3 protein expression based on CPTAC (**d**) and Human Protein Atlas **(e, f)** analyzes. Antibody, HPA006543; ***P < 0.001

Pan-cancer expression of CCT3 mRNA in a range of malignancies, displayed by the comparison between tumors and corresponding normal tissue counterparts, were investigated by using TCGA databases. CCT3 mRNA was found to be significantly upregulated in tumor tissues of 18 cancer species including LUAD (Fig. 2). The maximal log2 fold change (tumor vs. normal) of CCT3 expression was observed in cholangiocarcinoma (CHOL, 29.6), and that of LUAD was 27.5. However, CCT3 mRNA was also found to be downregulated in tumor tissues of kidney chromophobe (KICH) and kidney renal clear cell carcinoma (KIRC) (Fig. 2). On the other hand, scatter dot plot displaying RNA-seq expression levels from TCGA LUAD dataset also revealed that CCT3 mRNA expression was substantially higher in tumor than that in normal lung tissues (P = 2.287e-33, Normal = 59 and Tumor = 535) (Fig. 3a). This was further verified by the paired sample analysis of CCT3 expression in 57 LUAD cases and 57 matched normal cases (Fig. 3b; P = 2.2e-22). Intriguingly, our results showed that the *TP53* mutant LUAD cohort had significantly higher CCT3 mRNA expression than that of *TP53* non-mutant group. (Fig. 3c; P = 3.37 E-04, *TP53*-mutant = 233 and *TP53*-Nonmutant = 279). These results suggest that CCT3 mRNA is dysregulated in LUAD.

### Upregulation of CCT3 protein in LUAD tissues

The UALCAN portal of CPTAC dataset was used to explore the protein expression level of CCT3 in LUAD. As shown in Fig. 3d, LUAD tumor tissues had significantly higher level of CCT3 protein expression than normal lung tissues (P = 2.464e-18, Normal = 111 and Primary tumor = 111). The immunohistochemical results from the HPA database confirmed the differentiated protein expression of CCT3 by showing that the staining intensity of CCT3 in normal lung tissues was substantially lower than that in LUAD tissues (Fig. 3e and f). Thus, CCT3 mRNA and protein levels are both augmented in LUAD samples through comparative analyses across normal and tumor tissues.

### Dysregulated CCT3 is associated with sex, smoking and tumor staging of LUAD

**Fig. 4:**
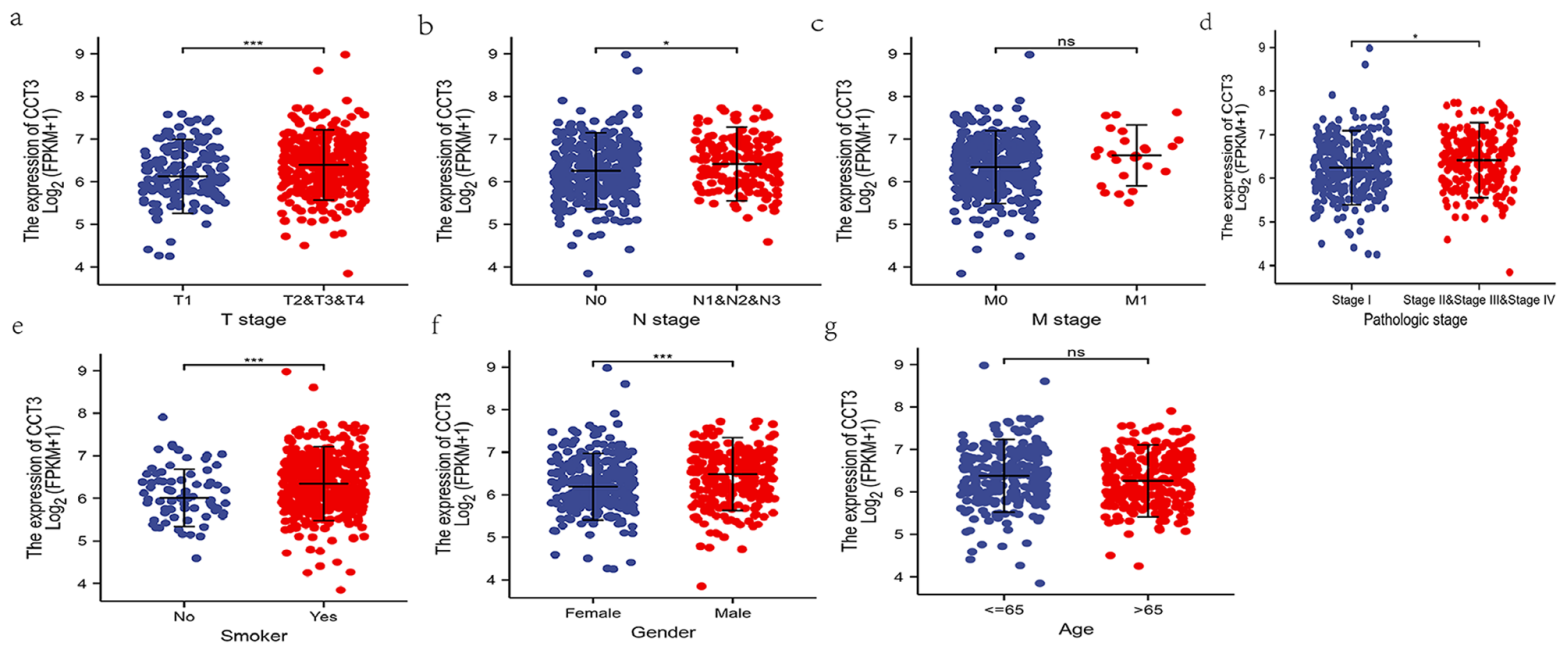
Association of CCT3 expression with clinicopathologic characteristics. CCT3 mRNA level was compared between T- **(a)**, N- **(b)** and M-categories **(c)**, pathologic stages **(d)**, smokers and non-smokers **(e)**, male and female **(f)**, and ≤ 65 and > 65 of LUAD patients. **(g)** *P < 0.05; ***P < 0.001; ns, no significance

The association between CCT3 expression and demographic and clinicopathological features were investigated in LUAD. TCGA’s data indicated that CCT3 expression is significantly increased in T2-T4 (*vs.* T1) and N1-N3 (*vs.* N0) of the TNM staging system, the pathological stage II - IV (*vs.* stage I), and in males and smokers. However, CCT3 expression has no significant difference between age groups (≤ 65 and > 65), and between M0 and M1 LUAD patients. (Fig. 4a-g). The logistic regression analysis confirmed the association between CCT3 and the demographic and clinicopathological features, with the exception of comparison between M0 and M1 stage (Odds Ratio (OR) = 2.16; P = 0.05) (Table 1). In the majority of statistical analyses, P = 0.05 (an alpha level of 0.05) is used as the cutoff value for significance. Collectively, these findings suggest that CCT3 expression is closely related to tumor progression, sex and smoking status of LUAD patients.

**Table 1:** Logistic regression analysis for the association between CCT3 and the demographic and clinicopathological features of LUAD

Characteristics	Total(N)	Odds Ratio (OR)	P value
T stage (T2&T3&T4 *vs.* T1)	532	2.35	0.001
N stage (N1&N2&N3 *vs.* N0)	519	2.24	0.010
M stage (M1 *vs.* M0)	386	2.16	0.050
Pathologic stage (Stage II &Stage III &Stage IV *vs.* Stage I)	527	1.75	0.024
Gender (Male *vs.* Female)	535	-2.22	0.005
Age (> 65 *vs.* <=65)	516	-2.15	0.153
Smoker (Yes *vs.* No)	521	-2.03	0.002

### Prognostic performance of CCT3 for LUAD

**Fig. 5:**
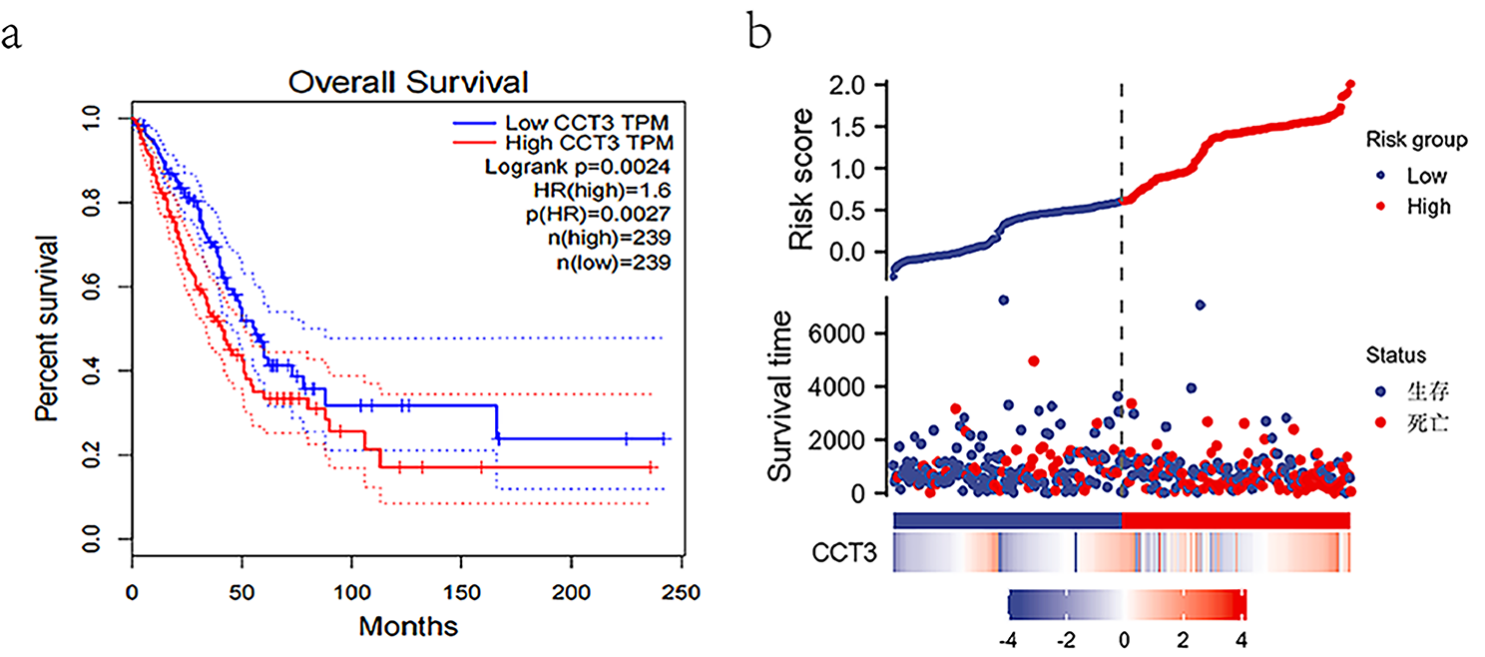
Prognostic prediction evaluation of CCT3 for LUAD. (**a)** Overall survival of CCT3 low and high LUAD patients showed by K-M survival plot. (**b)** Distribution of risk score (upper) and its association with survival time CCT3 expression (lower) according to the risk score model

**Fig. 6:**
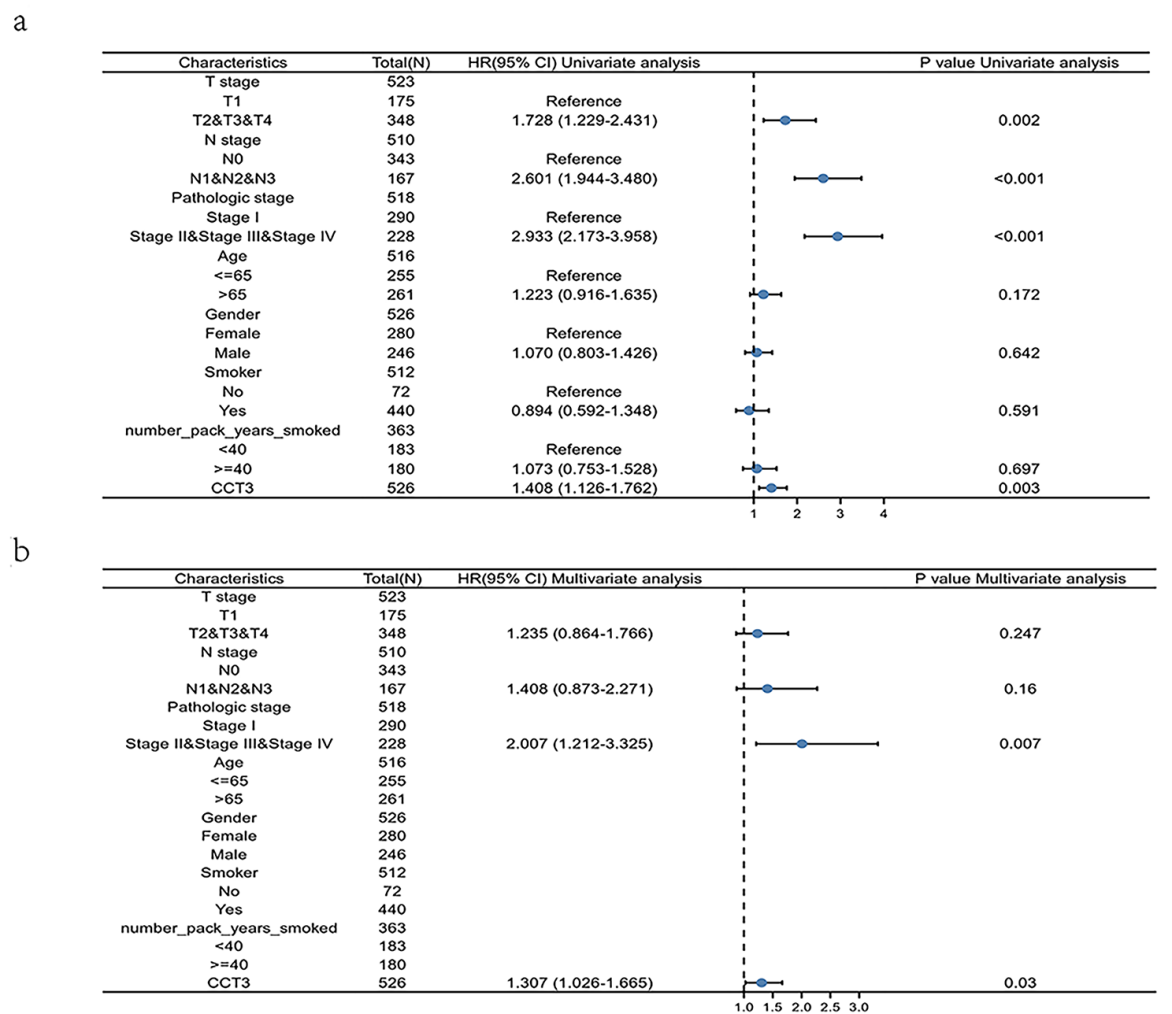
Univariate **(a)** and multivariate **(b)** COX regression analyzes of CCT3 expression in LUAD.

The Kaplan-Meier survival analysis showed that LUAD patients with higher expression of CCT3 mRNA (n = 239) had shorter overall survival (OS) as compared with lower CCT3 mRNA cohort (n = 239) (Fig. 5a; Log rank P = 0.0024). The hazard ratio (HR) in the higher versus lower CCT3 group is 1.6 (P = 0.0027). Also, the risk score plot confirmed the prognostic value of CCT3 for LUAD (Fig. 5b). CCT3 mRNA level, T- and N-categories and pathological staging were linked to LUAD OS in a univariate Cox hazard regression model (Fig. 6a). Furthermore, the multivariate Cox hazard regression analysis demonstrated the significant link of CCT3 expression and pathological stage with LUAD OS. (Fig. 6b). In conclusion, these findings suggest that CCT3 has excellent prognostic performance in LUAD and thus may be an independent prognostic factor for LUAD.

### Nomogram verification of CCT3 as prognostic factor for LAUD

**Fig. 7:**
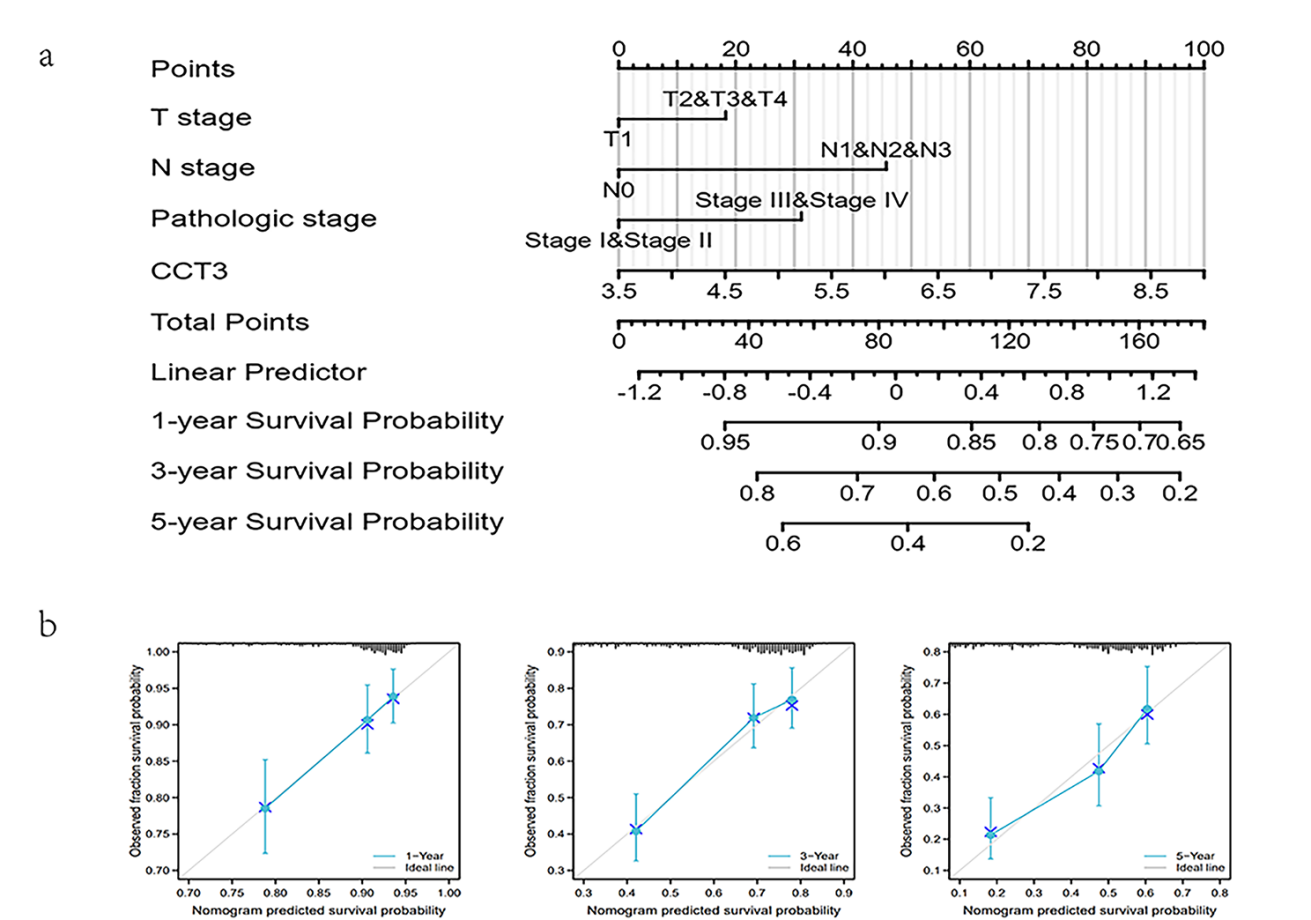
CCT3-related nomogram prognostic model for LUAD. (**a)** Construction of nomogram for both CCT3 and clinical characteristics to predict 1, 3, 5-year survival rates of LUAD. (**b)** Calibration curve of the nomogram predicting the probability of OS at 1, 3, and 5-year.

A nomogram prognostic model based on stratified expression of CCT3, T- and N-categories, and pathological staging was used to verify the predictive role of CCT3 for LUAD. The prognostic nomogram for OS in the primary cohort is shown in Fig. 7a and the C-index for OS prediction was 0.658 (95% CI, 0.633 to 0.682). The calibration plot for the probability of survival at 1, 3, and 5 years showed a good agreement between the prediction by nomogram and actual observation (Fig. 7b). Thus, the nomogram has the potential to be used as a valid model for predicting the survival of LUAD.

### Construction of gene and protein network of CCT3 in LUAD

**Fig. 8:**
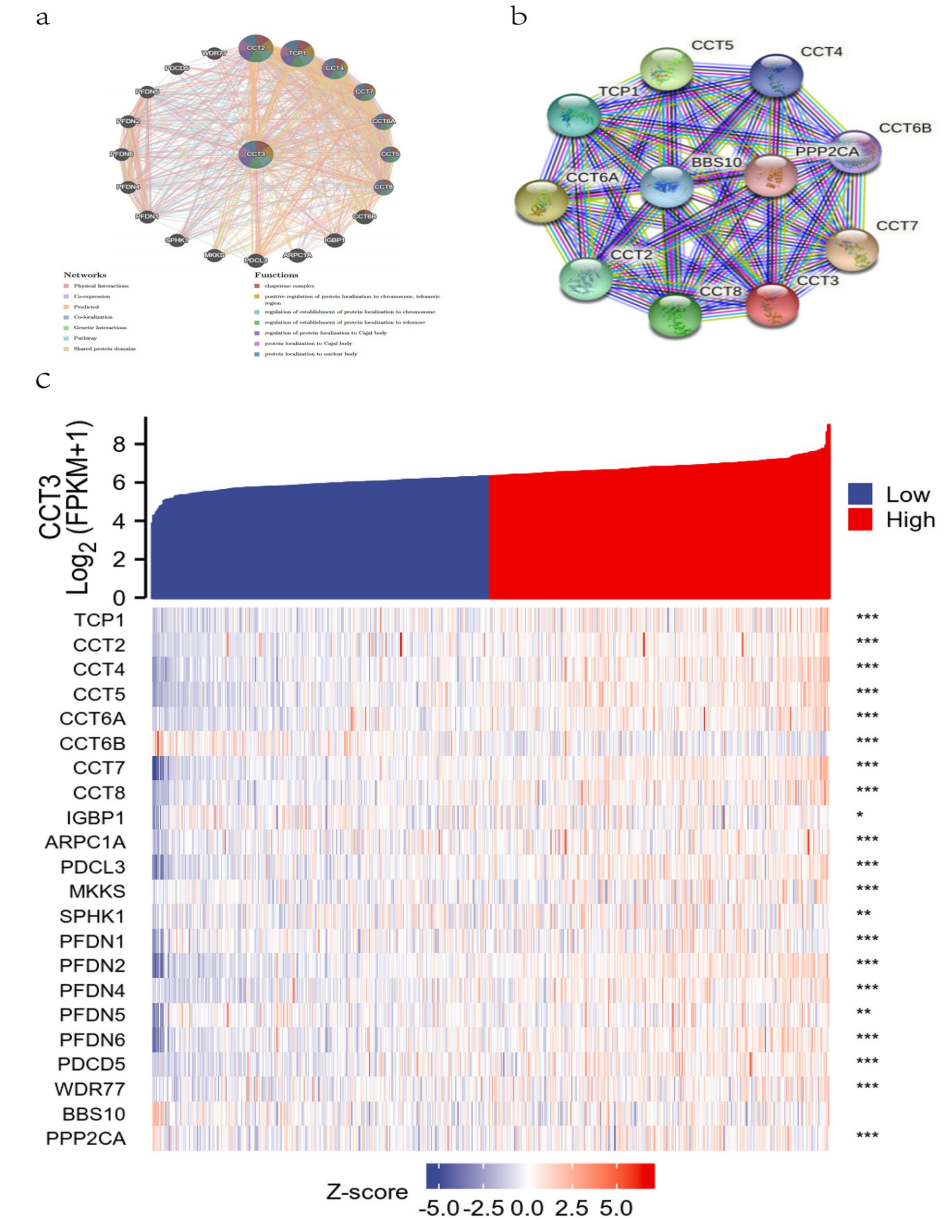
Interaction network of CCT3 and screening of Hub genes in LUAD. Genetic **(a)** and PPI **(b)** networks of CCT3 were constructed by GeneMANIA and String database, respectively. **(c)** Heatmap of CCT3-related hub genes in LUAD. *P < 0.05; **P < 0.01; ***P < 0.001

*CCT3*-related gene network was constructed by using data from GeneMANIA database that allows the user to color the network nodes by function (GO annotation). A total of 20 nodes around CCT3 were listed, including *CCT2*, *TCP1*, *CCT4*, *CCT7*, *CCT6A*, *CCT6B*, *CCT5*, *CCT8*, *IGBP1* (Immunoglobulin Binding Protein 1), *ARPC1A* (Actin Related Protein 2/3 Complex Subunit 1 A), *PDCL3* (Phosducin Like 3), *MKKS* (McKusick-Kaufman syndrome), *SPHK1* (Sphingosine Kinase 1), *PFDN* (Prefoldin) 1/4/6/2/5, *PDCD5* (Programmed Cell Death 5) and *WDR77* (WD Repeat Domain 77) (Fig. 8a). Next, the protein-protein interaction (PPI) predicted by the String database showed that 10 genes were involved in the network, including the above mentioned 8 subunits of CCT/TRiC complex, and BBS10 (Bardet-Biedl Syndrome 10) and PPP2CA (Protein Phosphatase 2 Catalytic Subunit Alpha) (Fig. 8b). We further analyzed the correlation between CCT3 and the total 22 related genes in LUAD and showed that 20 genes except *CCT6B* and BBS 10 were significantly correlated with CCT3 (Fig. 8c). Collectively, these findings established interaction network for CCT3 and depicted LUAD-specific protein-protein and genetic interaction networks to gain insight into the tumor-promoting effects of CCT3 in LUAD.

### Composition of CCT3-related immune infiltrates in LUAD

**Fig. 9:**
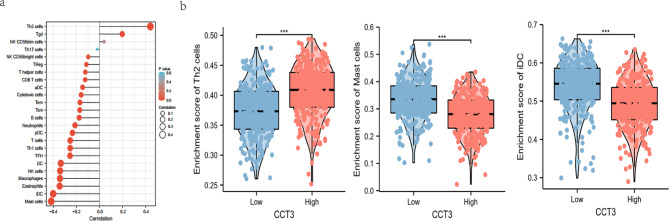
Correlations between CCT3 and the infiltrating immune cells in LUAD. CCT3-related infiltrating immune cells were predicted by the ssGSEA analysis **(a)**. **(b)** Comparisons of Th2 cells, Mast cells and iDC (screened by r > 0.4 or r < -0.4 between CCT3 high and low expression groups. ***P < 0.001

The composition analysis of infiltrating immune cells by ssGSEA (determined by r > 0.4 or r < -0.4) showed that CCT3 expression was positively correlated with the infiltrating Th2 cells (r = 0.442, p < 0.01) while negatively correlated with mast cells (r = -0.49, p < 0.01) and immature dendritic cells (iDCs, r = -0.401, p < 0.001) (Fig. 9a). Next, CCT3 expression was stratified by high (above median) and low (below median) mRNA levels, and significantly higher enrichment score of Th2 cells (P < 0.001) were observed in CCT3 high group whereas that of mast cells and iDCs (both P < 0.001) were observed both in CCT3 low group (Fig. 9b). Collectively, these findings demonstrated that dysregulation of CCT3 is characteristic of immune cell infiltrating in LUAD.

### GSEA enrichment analysis

**Fig. 10:**
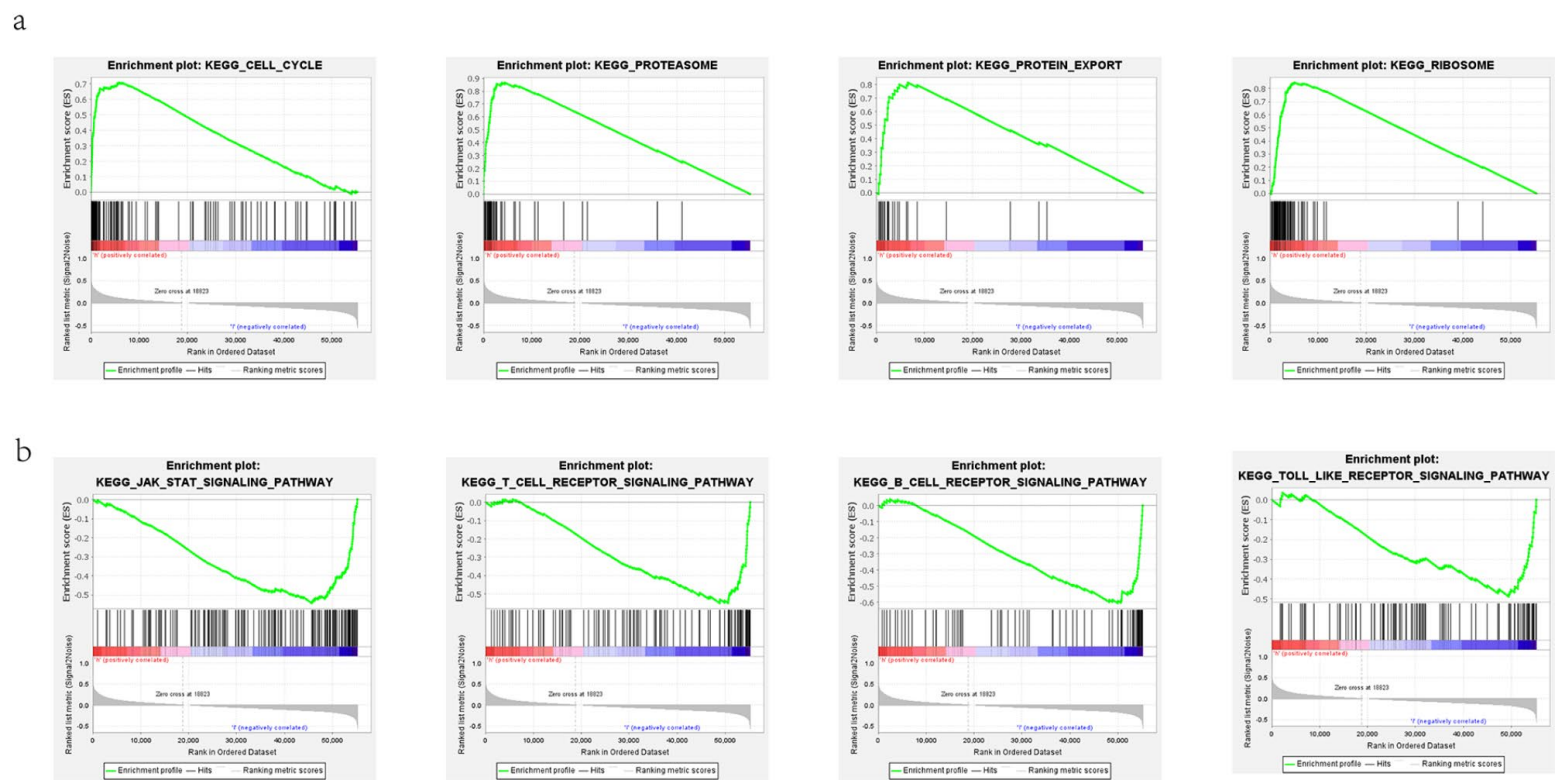
Clustered heatmap of CCT3-related KEGG pathway enrichment analysis in LUAD. **(a)** CCT3 high group. (**b)** CCT3 low group

By using GSEA computational method, pathway enrichment analysis was performed to identify CCT3-related signaling pathways with the threshold of p < 0.05 and FDR < 0.25. Of note, the cell cycle pathway, the protein export pathway, the proteasome pathway and the ribosome pathway were significantly enriched in CCT3 high group (Fig. 10a). In contrast, the signaling pathways including JAK-STAT, B cell receptor (BCR), T cell receptor (TCR) and toll like receptor (TLR) cascades were highly enriched in CCT3 low group (Fig. 10b). The enrichment score (ES), normalized enrichment score (NES), nominal P value, and false discovery rate (FDR) of these enriched pathways are shown in Table 2. Thus, these findings gain mechanistic insight into CCT3 effects in maintaining cellular proteostasis, especially proteins associated with cell cycle, and CCT3-related immunosuppression in LUAD.

**Table 2:** The KEGG Enrichment Pathways in CCT3 high and low groups

NAME	ES	NES	NOM p-value	FDR q-value
KEGG CELL CYCLE	0.71	2.35	0	0
KEGG PROTEIN EXPORT	0.82	2.24	0	0
KEGG PROTEASOME	0.87	2.16	0	0.001
KEGG RIBOSOME	0.85	1.75	0.014	0.028
KEGG JAK STAT SIGNALING PATHWAY	-0.54	-2.22	0	0.003
KEGG B CELL RECEPTOR SIGNALING PATHWAY	-0.61	-2.15	0.002	0.003
KEGG T CELL RECEPTOR SIGNALING PATHWAY	-0.55	-2.03	0.004	0.007
KEGG TOLL LIKE RECEPTOR SIGNALING PATHWAY	-0.49	-1.83	0.01	0.031

ES, enrichment score; NES, normalized enrichment score; NOM p-value: the nominal p value; FDR, False Discovery Rate

### ***CCT3***knockdown inhibited proliferation and promoted apoptosis of LUAD A549 cells

**Fig. 11:**
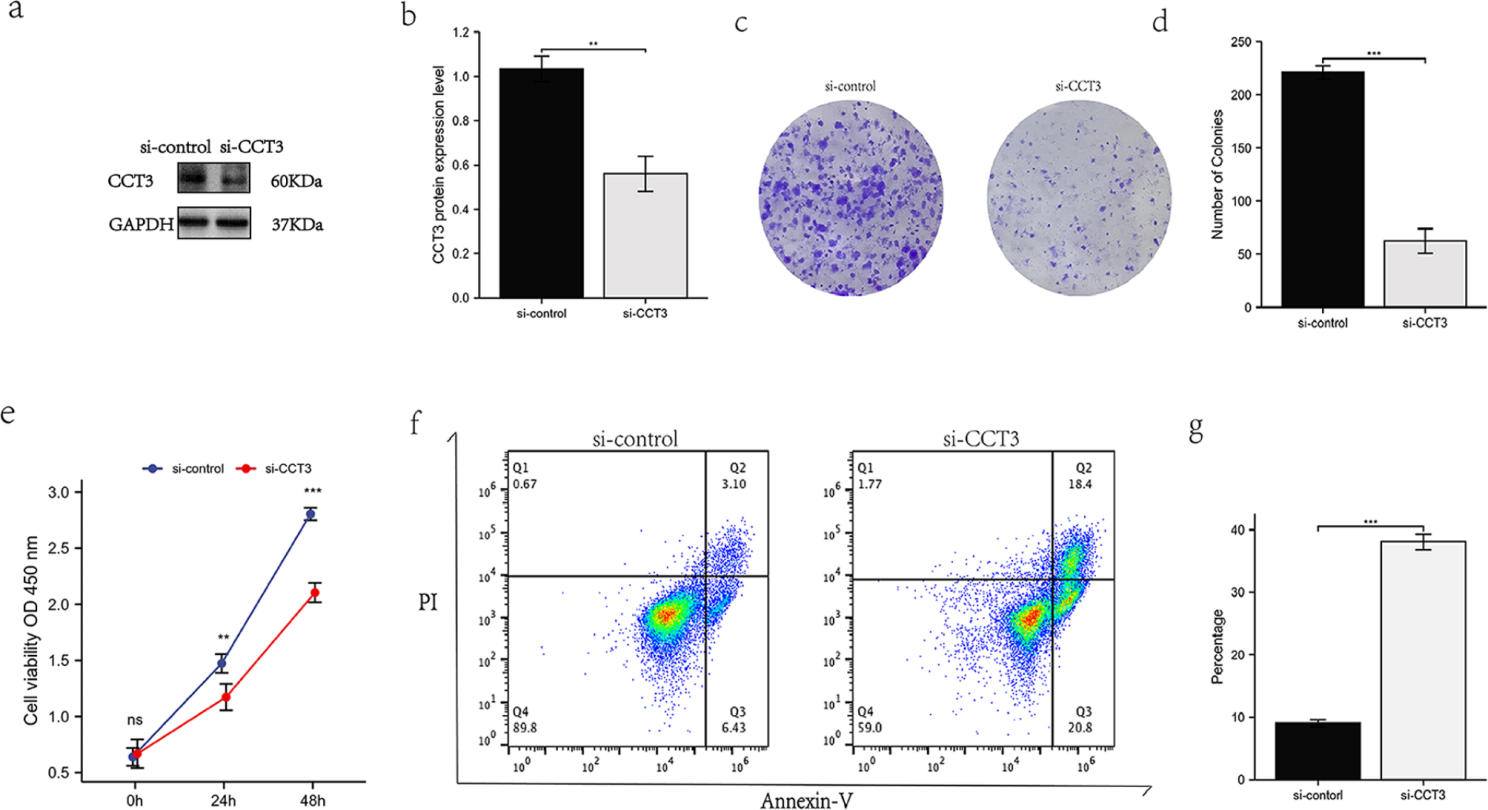
*CCT3* knockdown suppressed colony formation and cell proliferation but induced apoptosis of LUAD A549 cells. **(a, b)** Western blotting **(a)** and band intensity **(b)** analyzes of small interfering RNA mediated CCT3 knockdown in A549 cells, GAPDH was used as the loading control. **(c, d)** Representative colony formation **(c)** and comparisons of colony counts **(d)** between si-CCT3 and si-Control cells. **(e)** The proliferation of si-CCT3 and si-Control A549 cells were determined by WST-8/Cell Counting Kit-8 at 0, 24 and 48 h after cell culturing. **(f, g)** Representative flow cytometry **(f)** and comparisons of percentages of total Annexin-V^+^ cells **(g)** between si-CCT3 and si-Control A549 cells. Data are representative of three independent experiments. **P < 0.01, ***P < 0.001, ns, no significance

Western blotting assay showed that siRNAs effectively downregulate CCT3 expression in A549 cells (Fig. 11a and b). Si-CCT3 A549 cells had substantially decreased numbers of colony formation (P < 0.001) (Fig. 11c and d) and significantly reduced cell proliferation (P < 0.01 at 24 h and P < 0.001 at 48 h) as compared with Si-control (Fig. 11e). On the other hand, the representative flow cytometry profile (Fig. 11f and g) showed that 39.2% Si-CCT3 A549 cells were Annexin-V positive, compared to that of 9.53% in corresponding Si-control cells. The statically apoptotic rates (%) between Si-CCT3 and Si-control A549 cells were 38.067 ± 1.266 and 9.200 ± 0.554, respectively (P < 0.001) (Fig. 11g). Taken together, these results confirmed bioinformatics-based findings by demonstrating the oncogenic role of CCT3 in promoting proliferation and suppressing apoptosis of LUAD cells.

## Discussion

Being a member of group II chaperonins, CCT/TRiC focuses on maintaining cellular proteostasis of the major cytoskeletal proteins including tubulins and actins [5]. It has been widely reported that CCT3, a subunit of CCT/TRiC complex, has tumor-promoting effects in various malignancies including LUAD [6–10]. In this study, we provide further multi-scale dataset evidence that CCT3 mRNA is significantly upregulated in 18 cancer species while downregulated in tumor tissues of kidney chromophobe (KICH) and kidney renal clear cell carcinoma (KIRC). The maximal log2 fold change (tumor *vs.* normal) of CCT3 mRNA expression was observed in cholangiocarcinoma (CHOL, 29.6), and that of LUAD was 27.5. Furthermore, both CPTAC and HPA database analyses showed the overexpression of CCT3 protein in LUAD tumor tissues. Thus, the pan-cancer dysregulation of CCT3 occurs in an opposite direction and future research focused on CCT3 downregulation in renal neoplasm will facilitate a better understanding of CCT3 effects in malignancy. The substantial upregulation of CCT3 observed in this study is consistent with multiple lines of evidence from LUAD tissues and cells [7–10]. Furthermore, CCT3 dysregulation in LUAD makes it a potential therapeutic target since CCT3 has been reported to promote cell proliferation, cell-cycle progression, cisplatin resistance, ferroptosis/apoptosis and ATP production of LUAD cells [7–9].

Intriguingly, our results showed that the *TP53* mutant LUAD cohort had significantly higher CCT3 expression than that of *TP53* non-mutant group. *TP53* is a tumor suppressor gene encodes for P53 protein, which regulates the expression of series target genes involved in DNA repair, cell-cycle arrest and apoptosis. Somatic *TP53* mutations drive oncogenesis and occur in almost all types of cancers at rates from 30-50% [27], and are more frequent in NSCLC patients with tobacco smoking [28]. Furthermore, *TP53* mutations have close association with advanced stage or cancer subtypes with aggressive behavior. Thus, the association between CCT3 expression and *TP53* mutations hints an oncogenic role of CCT3 and its association with development and progression of LUAD.

Indeed, our next findings support the notion by showing that CCT3 expression is significantly upregulated in T2-T4 (*vs.* T1), N1-N3 (*vs.* N0), the pathological stage II-stage IV (*vs.* stage I) patients, and in males and smokers. Accordingly, CCT3 mRNA level was significantly linked to LUAD OS both in univariate and multivariate Cox hazard regression models. The Kaplan-Meier survival analysis, together with the risk score and the nomogram prognostic models verified the predictive role of CCT3 for LUAD. However, CCT3 expression has no significant difference between age groups (≤ 65 and > 65), and between M0 and M1 LUAD patients. Being a multi-stage process, distant metastasis begins with invasion of cancer cells into surrounding tissue, followed by intravasation, vascular transit, extravasation, and formation of site-specific lesions [29]. We propose that CCT3 is not a determinator for distant metastasis although many of these steps require cell motility driven by cycles of actin polymerization. On the other hand, it is very likely that higher CCT3 observed in males is derived from smoking since males tend to use all tobacco products at higher rates than females. Thus, these findings suggest that CCT3 may be an independent prognostic factor for LUAD.

The other 7 subunits consisting of CCT/TRiC, CCT2, CCT1 (TCP1), CCT4, CCT7, CCT6A, CCT5 and CCT8, establish genetic interactions and co-expression network with CCT3. In contrast to ubiquitous expression of CCT6A, CCT6B mRNA is detected only in testis across human and murine genomes [30]. Besides, 12 nodes around CCT3 were listed in GeneMANIA and two genes, BBS10 and PPP2CA, were found in String database (Fig. 8a, b). Of the 14 genes, IGBP1 (also known as α4 protein) contributes to assembly and stability of protein phosphatase 2 A (PP2A) that is one of the most conserved phospho-Ser/Thr phosphatases in eukaryotic cells. Genetic deletion of α4 significantly suppressed PP2A-dependent dephosphorylation of P53, ATM (ataxia-telangiectasia mutated) and histone H2AX that are required to DNA [31]. The *PPP2CA* gene also plays a vital role in the activity of PP2A by encoding the α subtype of the catalytic subunit and selecting the PP2A regulatory subunit [32]. ARPC1A is an isoform of ARPC1, which consists with other 6 subunits to form the seven-subunit actin-related proteins-2/3 (ARP2/3) complex. ARP2/3 is unique in its capability to organize filaments into branched networks [33]. PDCL3 acts as co-chaperone during CCT-assisted protein folding of the cytoskeletal components including actin and β-tubulin [34]. The *MKKS* (McKusick-Kaufman syndrome) gene encodes a 570-amino acid polypeptide with weak but significant similarity to group II chaperonins. Mutations in the *MKKS* gene also cause Bardet-Biedl syndrome (BBS), thus MKKS also known as BBS6. The chaperonin-like BBS proteins, for example, MKKS/BBS6 and BBS10, along with CCT/TRiC protein to form a stable complex, the BBSome, which is proposed to function in vesicle trafficking [35]. However, no significant correlation was observed between CCT3 and BBS10 in LUAD. As a lipid kinase, SPHK1 phosphorylates sphingosine to bioactive sphingosine-1-phosphate (S1P) that is critical for cell motility, cytoskeletal organization, cell growth and response to stress [36]. Prefoldin (PFDN), an ATP-independent chaperone is essential to prevent toxic conformations and ensure effective cellular proteostasis by escorting misfolded or non-native proteins to TRiC/CCT [37]. Previous study has shown that PDCD5 enhance the stability of p53 by antagonizing E3 ubiquitin-protein ligase Mdm2-induced p53 ubiquitination, nuclear export and proteasomal degradation [38]. Finally, WDR77, also known as methylosome protein 50 (MEP50), is a core component of a transmethylase complex with the protein arginine methyltransferase 5 (PRMT5), enabling the methylation of target proteins such as P53 and histone H4 [39]. The GSEA enrichment analysis further supported these results by showing that the cell cycle pathway, the protein export pathway, the proteasome pathway and the ribosome pathway were significantly enriched in CCT3 high group. Collectively, these findings establish that CCT3, or rather CCT/TRiC chaperonin, collaborates with other chaperons to facilitate the assembly and stability of proteins involved in proteostasis of cytoskeletal filaments, DNA repair and protein methylation.

The ssGSEA analysis highlighted positive correlation of the infiltrating Th2 cells versus negative correlation of mast cells and iDCs with CCT3 in LUAD. CCT3-mediated Th2 deviation in tumor microenvironment may link to a worse outcome for LUAD, since Th2 cytokines IL-4 IL-5, IL-9 and IL-13 trigger the differentiation of tumor-associated M2 macrophages [40]. Mast cells act through direct cell-to-cell interactions or secretion of biologically active factors to shape tumor environment [41]. Our observation suggests a protective role of mast cells in LUAD and further experimental study is needed to clarify the underlying mechanisms. In a well-established tumor, tumor-infiltrating dendritic cells are suppressed by cancer cells and therefore stay in the favorable immature state [42]. Such immature DCs (iDCs) have decreased antigen-presenting capabilities and canonical immature phenotypes, however, their endocytic activity is also suppressed. These findings are consistent with evidences from GSEA analyses that the JAK-STAT, BCR, TCR and TLR signaling pathways were highly enriched in CCT3 low group. Thus, dysregulation of CCT3 is characteristic of immune cell infiltration in tumor microenvironment and a better understanding of CCT-related immunosuppression will be conducive to immunotherapy for LUAD. Our experimental results performed by CCT3 knockdown showed that Si-CCT3 A549 cells had substantially decreased colony formation and cell proliferation but increased apoptosis as compared with si-Control. These are consistent with recently published data acquired from both H1299 and A549 cells [10]. The investigators further highlighted CCT3 effects on proliferation, showing that CCT3 knockdown reduced glycolysis-dependent ATP production and eukaryotic translation initiation factor 3 (EIF3G) synthesis and thereby impairing protein translation in LUAD cells. This study broadens our understanding by establishing CCT3-related signaling and PPI network apart from its regulation on proteostasis of cytoskeletal filaments. Also, these findings agree well with previous research in other cancers [43–45]. Taken together, evidence has accumulated indicating the oncogenic role of CCT3 in LUAD.

## Conclusion

In this study, we provide novel insights into the dysregulation of CCT3 and its excellent prognostic performance in LUAD. The oncogenic role of CCT3 was investigated by establishing CCT3-related genetic and PPI networks, enrichment of signaling pathways and infiltration of immune cells in the tumor microenvironment of LUAD. Besides, we conducted in vitro experiments to verify CCT3 effects on LUAD cells. Our findings suggest that CCT3 is a critical negative immunoregulatory factor and therapeutic target for LUAD.

## Electronic supplementary material

Below is the link to the electronic supplementary material.

Supplementary Material 1

## Data Availability

The datasets analyzed during the current study are available in the TCGA repository, [The Cancer Genome Atlas Program - NCI]. .
