# Supplementary figures and images for: Integrated analyzes identify CCT3 as a modulator to shape immunosuppressive tumor microenvironment in lung adenocarcinoma
Source: BMC Cancer. 2023 Mar 14;23:241. doi: 10.1186/s12885-023-10677-w (PMC10012614; doi:10.1186/s12885-023-10677-w)

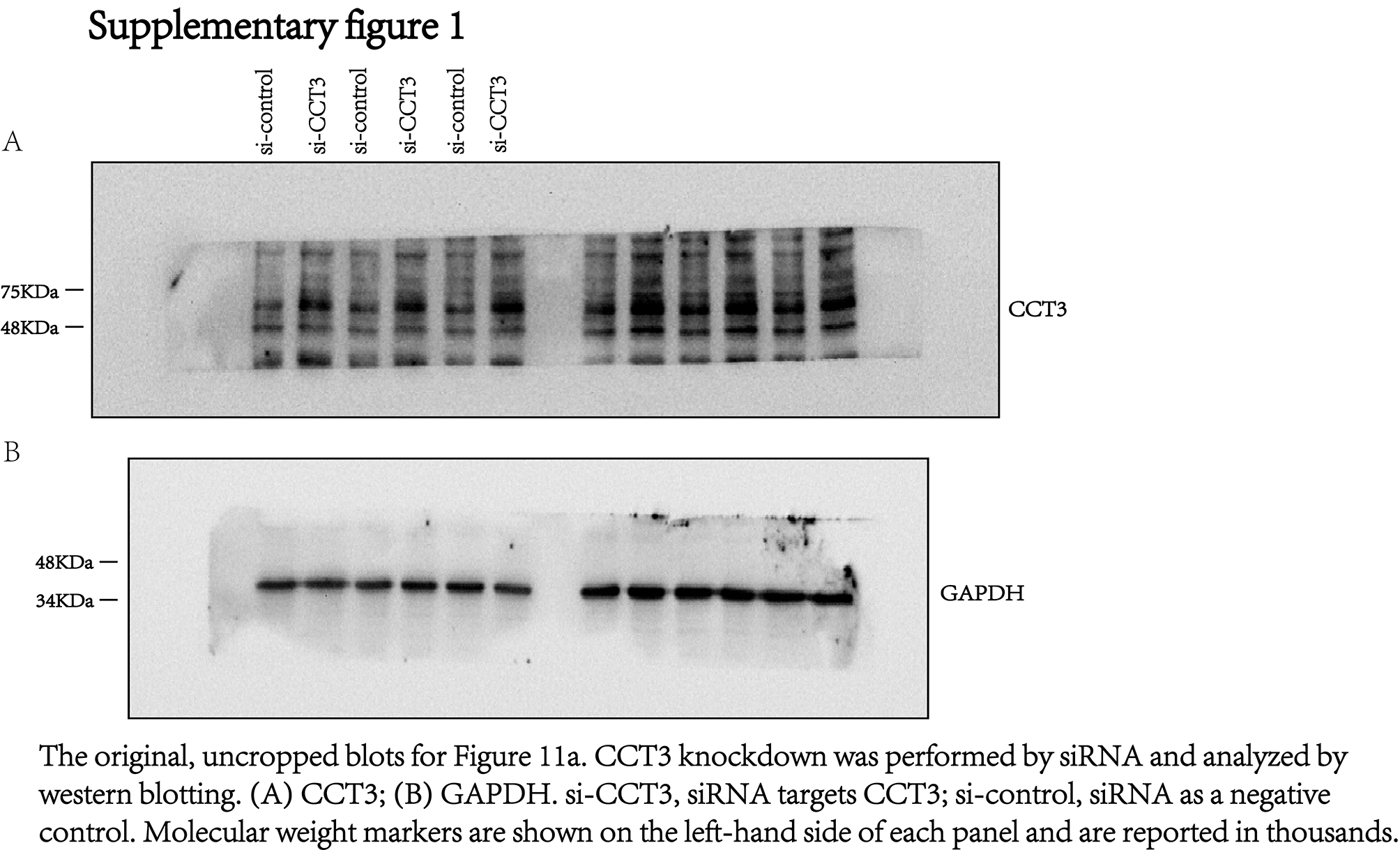

Supplement: Supplementary file 1 — Supplementary Material 1 [file 12885_2023_10677_MOESM1_ESM.tif]
